# Regional brain tissue changes in patients with cystic fibrosis

**DOI:** 10.1186/s12967-021-03092-x

**Published:** 2021-10-09

**Authors:** Bhaswati Roy, Marlyn S. Woo, Susana Vacas, Patricia Eshaghian, Adupa P. Rao, Rajesh Kumar

**Affiliations:** 1 Department of Anesthesiology and Perioperative Medicine, University of California at Los Angeles, Los Angeles, CA 90095 USA; 2 Department of Pediatric Pulmonology, University of California at Los Angeles, Los Angeles, CA 90095 USA; 3 Department of Medicine, University of California at Los Angeles, Los Angeles, CA 90095 USA; 4 Department of Medicine, Keck School of Medicine, University of Southern California, Los Angeles, USA; 5 Department of Radiological Sciences, University of California at Los Angeles, Los Angeles, CA 90095 USA; 6 Department of Bioengineering, University of California at Los Angeles, Los Angeles, CA 90095 USA; 7 The Brain Research Institute, University of California at Los Angeles, Los Angeles, CA 90095 USA

**Keywords:** Cognition, Mood, Gray matter density, T2-relaxometery, Magnetic resonance imaging

## Abstract

**Background:**

Cystic fibrosis (CF) patients present with a variety of symptoms, including mood and cognition deficits, in addition to classical respiratory, and autonomic issues. This suggests that brain injury, which can be examined with non-invasive magnetic resonance imaging (MRI), is a manifestation of this condition. However, brain tissue integrity in sites that regulate cognitive, autonomic, respiratory, and mood functions in CF patients is unclear. Our aim was to assess regional brain changes using high-resolution T1-weighted images based gray matter (GM) density and T2-relaxometry procedures in CF over control subjects.

**Methods:**

We acquired high-resolution T1-weighted images and proton-density (PD) and T2-weighted images from 5 CF and 15 control subjects using a 3.0-Tesla MRI. High-resolution T1-weighted images were partitioned to GM-tissue type, normalized to a common space, and smoothed. Using PD- and T2-weighted images, whole-brain T2-relaxation maps were calculated, normalized, and smoothed. The smoothed GM-density and T2-relaxation maps were compared voxel-by-voxel between groups using analysis of covariance (covariates, age and sex; SPM12, p < 0.001).

**Results:**

Significantly increased GM-density, indicating tissues injury, emerged in multiple brain regions, including the cerebellum, hippocampus, amygdala, basal forebrain, insula, and frontal and prefrontal cortices. Various brain areas showed significantly reduced T2-relaxation values in CF subjects, indicating predominant acute tissue changes, in the cerebellum, cerebellar tonsil, prefrontal and frontal cortices, insula, and corpus callosum.

**Conclusions:**

Cystic fibrosis subjects show predominant acute tissue changes in areas that control mood, cognition, respiratory, and autonomic functions and suggests that tissue changes may contribute to symptoms resulting from ongoing hypoxia accompanying the condition.

## Introduction

Cystic fibrosis (CF) is a progressive genetic disorder predominately affecting lungs, liver, and the pancreas and intestine exocrine glands. Approximately 1000 new CF cases are diagnosed each year, totaling more than 30,000 people in the United States and 70,000 worldwide [1, 2]. CF is caused by mutations in the CF transmembrane conductance regulator (CFTR) gene, which is located on chromosome 7 and provides the synthesis of the CFTR protein that is responsible for the transport of chloride ion at the cell membrane level to regulate salt, fluid absorption, and secretion, and broadly classified into I–VI classes based on their effects on the CFTR protein [3, 4]. The CFTR gene mutation can cause the CFTR protein to malfunction and affect multiple organs and systems, including lungs, upper respiratory tract, gastrointestinal tract, pancreas, liver, sweat glands, and genitourinary tract [5], and may also affect brain.

Although CFTR mutations are present at birth, sometimes there are delayed diagnosis due to late onset of symptoms. Due to implementation of newborn screening program in recent years, 92.3% of CF diagnoses are among less than 6-months old [2]. The clinical features of CF patients predominantly include, respiratory, digestive, and reproductive disorders, with evidence of disease progression in digestive and respiratory systems can be observed mostly by 6 months of age, worsening of lung function from 6 months to 1 year of age, and accumulation of lung damage by 1 year of age [6]. Other symptoms include poor weight gain/growth, persistent cough, shortness of breath, and repeated lung infections. In addition, CF patients with clinically stable severe lung disease show impaired neurocognitive functions, including cognitive and mood deficits, autonomic issues, and daytime sleepiness [7], and disease exacerbation further worsens their neurobehavioral performance [8]. High rates of anxiety and depression found in CF lead to non-adherence of prescribed treatment, affecting health outcomes and health related quality of life [9]. Such psychological, including mood and cognitive functions, and autonomic deficits [10, 11] might result from tissue dysfunction in multiple brain regions; however, there are no previous studies examining brain changes in CF patients.

Subtle brain tissue changes are often challenging to visualize on routine brain magnetic resonance imaging (MRI), including T1-weighted and T2-weighted imaging. High-resolution T1-weighted imaging based voxel-based morphometry (VBM), and T2-relaxometry based on proton density (PD)- and T2-weighted imaging can be used to examine subtle brain tissue changes. VBM procedures can exhibit localized gray matter (GM) density that reflects the proportion of GM relative to other tissue types within an examined region. However, T2-relaxometry measures free-water content within the tissue [12] by acquiring a series of images at different echo times, and has the potential to detect brain tissue microstructural changes, with higher specificity than conventional MRI. Immuno-histochemical evidence shows that decreased T2-relaxation values are associated with increased glial activation [13], and reduced T2-relaxation values emerged in bipolar disorder [14, 15], and spinocerebellar ataxia type 3 [16]. In addition, the main etiologies of increased T2-relaxation values in the brain are vasogenic edema, demyelination, gliosis, or neuronal loss [17–19], observed in tumor [20], chronic epilepsy [21], congenital central hypoventilation syndrome [22], traumatic brain injury [23], and multiple sclerosis [17]. Such MRI techniques are simple and rapid, utilizes data acquired from routine T1-weighted, proton-density, and T2-weighted imaging, and can be implemented on standard clinical MR systems, and the quantitative measures make them advantageous in examining brain tissue integrity.

Average survival of CF patients has improved recently, and this improvement is due to the advancement in treatment, emphasis on early diagnosis, as well as effective differential disease management, though there is still no cure for the disease. In order to increase the life span and life quality of CF individuals, detection of brain changes are of utmost importance. Identifying the structural brain changes associated with cognitive and mood deficits in CF patients may provide new insights into healthcare management and long-term clinical strategies.

Our study aimed to examine regional GM density changes, as well as tissue changes using T2-relaxometry procedures in CF patients over healthy controls. Based on the severity of psychological and autonomic changes exhibited in CF patients, we hypothesized that GM density and T2-relaxation values would differ from healthy population, indicating brain damage, in autonomic, mood, and cognition control areas.

## Materials and methods

### Subjects

This is a cross-sectional, comparative study of five CF patients (mean age ± SD, 29.7 ± 3.7 years; male, 3) recruited from the University of California Los Angeles (UCLA) Adult Cystic Fibrosis Center and 15 control subjects (mean age ± SD, 33.9 ± 4.5 years; male, 10) recruited through advertisements at the UCLA campus and Los Angeles area. All study procedures were followed in accordance with institutional guidelines, and the study was approved by the UCLA Institutional Review Board (IRB # 14-000967). Subjects were fully informed about the study procedures and provided written informed consent prior to data collection. CF patients were confirmed for CF genotype, were with mutation class I–III, and had mild to moderate CF lung disease. None of the CF patient underwent lung transplant, were not on any steroid therapy, and their oxygen saturation at rest was > 94% on room air. CF patients with history of stroke, seizure disorder, or head trauma, diagnosed psychiatric disease (clinical depression, schizophrenia, manic-depressive), airway or chest deformities that would interfere with breathing were excluded from the study. Control subjects were healthy, with no sleep disturbances, neurological or cardiovascular issues that would introduce brain damage, or drug dependency that would modify brain tissue.

### Assessment of depression and anxiety

All CF and control subjects were assessed for anxiety and depression using the Beck anxiety inventory (BAI) and the Beck depression inventory (BDI-II), respectively [24, 25]. The BAI and BDI-II inventories are self-administered questionnaires, composed of 21 multiple-choice questions (each question score ranged 0–3), with total scores ranging from 0 to 63 based on symptom severity [24, 25].

### Cognition examination

CF and control subjects underwent for cognition evaluation using the Montreal Cognitive Assessment (MoCA) [26]. The MoCA test was used for rapid evaluation of various cognitive domains, including attention and concentration, executive functions, memory, language, visuo-constructional skills, conceptual thinking, calculations, and orientation. A score < 26 was considered abnormal [26].

### Magnetic resonance imaging

All brain imaging studies were performed in a 3.0-Tesla MR scanner (Magnetom Tim-Trio and Prisma Fit, Siemens, Erlangen, Germany). We used foam pads on either side of the head to minimize head motion. Proton density (PD) and T2-weighted images were acquired using a dual-echo turbo spin-echo sequence in the axial plane [repetition time (TR) = 10,000 ms; echo-time (TE1, TE2) = 12, 123/124 ms; flip angle (FA) = 130°; matrix size = 256 × 256; field-of-view (FOV) = 230 × 230 mm; slice thickness = 3.5 mm; inter-slice gap = no]. Two high-resolution T1-weighted images were collected using a magnetization prepared rapid acquisition gradient-echo (MPRAGE) sequence (TR = 2200 ms; TE = 2.3/2.4 ms; inversion time = 900 ms; FA = 9°; matrix size = 320 × 320; FOV = 230 × 230 mm; slice thickness = 0.9 mm; number of slices = 192).

### Data processing

We used the statistical parametric mapping package SPM12 (Wellcome Department of Cognitive Neurology, UK; http://www.fil.ion.ucl.ac.uk/spm/), and MATLAB-based (The MathWorks Inc, Natick, MA) custom software to process MRI data. Also, we used the MRIcroN software to visualize images.

#### Visual examination

High-resolution T1-weighted, PD-weighted, and T2-weighted images of CF and control subjects were examined for any visible brain changes, including cystic lesions, infarcts, tumors, or other types of brain lesions. All images were also assessed for motion-related or any other imaging artifacts before GM density and T2-relaxation calculations.

#### Calculation of GM density

Both high-resolution T1-weighted image series were realigned to remove any potential variations between scans, and averaged to improve signal-to-noise ratio. The averaged images were segmented into GM, white matter, and cerebrospinal fluid tissue types, using the Diffeomorphic Anatomical Registration through Exponentiated Lie algebra algorithm (DARTEL) toolbox [27], and created flow fields and template images. The flow fields and final template images were normalized to Montreal Neurological Institute (MNI) space (unmodulated, re-sliced to 1 × 1 × 1 mm^3^) and smoothed with a Gaussian filter (8 mm kernel).

#### Calculation of T2-relaxation

Using PD and T2-weighted images, whole-brain pixel-by-pixel T2-relaxation values were calculated [22, 28]. We calculated the average noise level outside the brain tissue from PD- and T2-weighted images, and was used as a noise threshold to exclude non-brain areas. The same noise threshold was used for the PD and T2-weighted images in all subjects. The following equation was used to calculate T2-relaxation values [22, 28, 29]:

$${\text{T}}_{2} = \frac{{\left( {{\text{TE}}_{2} - {\text{TE}}_{1} } \right)}}{{\ln \left( {\frac{{{\text{SI}}_{1} }}{{{\text{SI}}_{2} }}} \right)}},$$

where TE_1_ and TE_2_ are the echo-times for PD and T2-weighted images, and SI_1_, SI_2_ denote PD and T2-weighted images signal intensities, respectively. Whole-brain T2-relaxation maps were generated from each voxel value. T2-relaxation maps were normalized to the standard MNI space and smoothed using a Gaussian filter (8 mm).

### Statistical analyses

We used the statistical package for social sciences (SPSS^®^ v26) for data analyses. The independent samples t-tests were used to examine the demographic and clinical characteristics with continuous variables, and the Chi-square tests to assess categorical variables between CF and control subjects. The MoCA, BDI-II, and BAI scores were examined with analysis of covariance (ANCOVA; covariates; age and sex). A p value < 0.05 was considered statistically- significant.

We performed whole-brain voxel-based analyses procedures to examine regional brain changes between CF and control subjects. For assessment of regional brain GM density and tissue changes, the normalized and smoothed whole-brain GM density and T2-relaxation maps were compared voxel-by-voxel between groups using ANCOVA, with age and sex as covariates [SPM12; p < 0.001; uncorrected; minimum extended cluster size, 10 voxels]. The extended cluster size 10 was chosen to avoid brain sites with less than 10 voxels appearing as a cluster. Brain clusters with significant GM density and T2-relaxation value differences between CF and control subjects were overlaid onto the normalized mean anatomical images for structural identification.

Regional brain GM density and T2-relaxation values were calculated from region of interest (ROI) analyses and examined for significant magnitude differences between CF and control subjects.

## Results

### Subject characteristics

Demographic and clinical variables of CF and control subjects are summarized in Table 1. No significant difference in age (p = 0.08), sex (p = 0.79), or body mass index (p = 0.33) appeared between the groups.

**Table 1:** Demographics and other variables of CF and control subjects

Variables	CF (n = 5) Mean ± SD	Controls (n = 15) Mean ± SD	p-values
Age (years)	29.7 ± 3.7	33.9 ± 4.5	0.08
Sex [male] (%)	3 (60%)	10 (67%)	0.79
BMI (kg/m^2^, mean ± SD)	22.0 ± 0.8	23.8 ± 3.9	0.33
BAI	7.8 ± 4.4	1.7 ± 4.2	0.02
BDI-II	5.0 ± 3.0	1.3 ± 2.8	0.03
Total MoCA scores	26.5 ± 1.5	28.2 ± 1.4	0.04
MoCA: visuospatial	4.1 ± 0.6	4.8 ± 0.5	0.02
MoCA: naming	3.0 ± 0.0	3.0 ± 0.0	–
MoCA: attention	5.9 ± 0.8	5.5 ± 0.8	0.34
MoCA: language	2.4 ± 0.5	2.8 ± 0.4	0.17
MoCA: abstraction	1.6 ± 0.5	2.0 ± 0.4	0.13
MoCA: delayed recall	4.1 ± 1.0	4.2 ± 1.0	0.85
MoCA: orientation	5.7 ± 0.4	5.9 ± 0.3	0.30

*SD* standard deviation, *BMI* body mass index, *BDI-II* Beck depression inventory II, *BAI* Beck anxiety inventory, *MoCA* Montreal cognitive assessment

### Mood and cognitive scores

The BDI-II and BAI scores were significantly higher in CF over control subjects (Table 1). The total MoCA scores were significantly lower in CF as compared to control subjects (p = 0.04), and the visuospatial was the most affected sub-scale (p = 0.02).

### Regional GM density changes

**Fig. 1:**
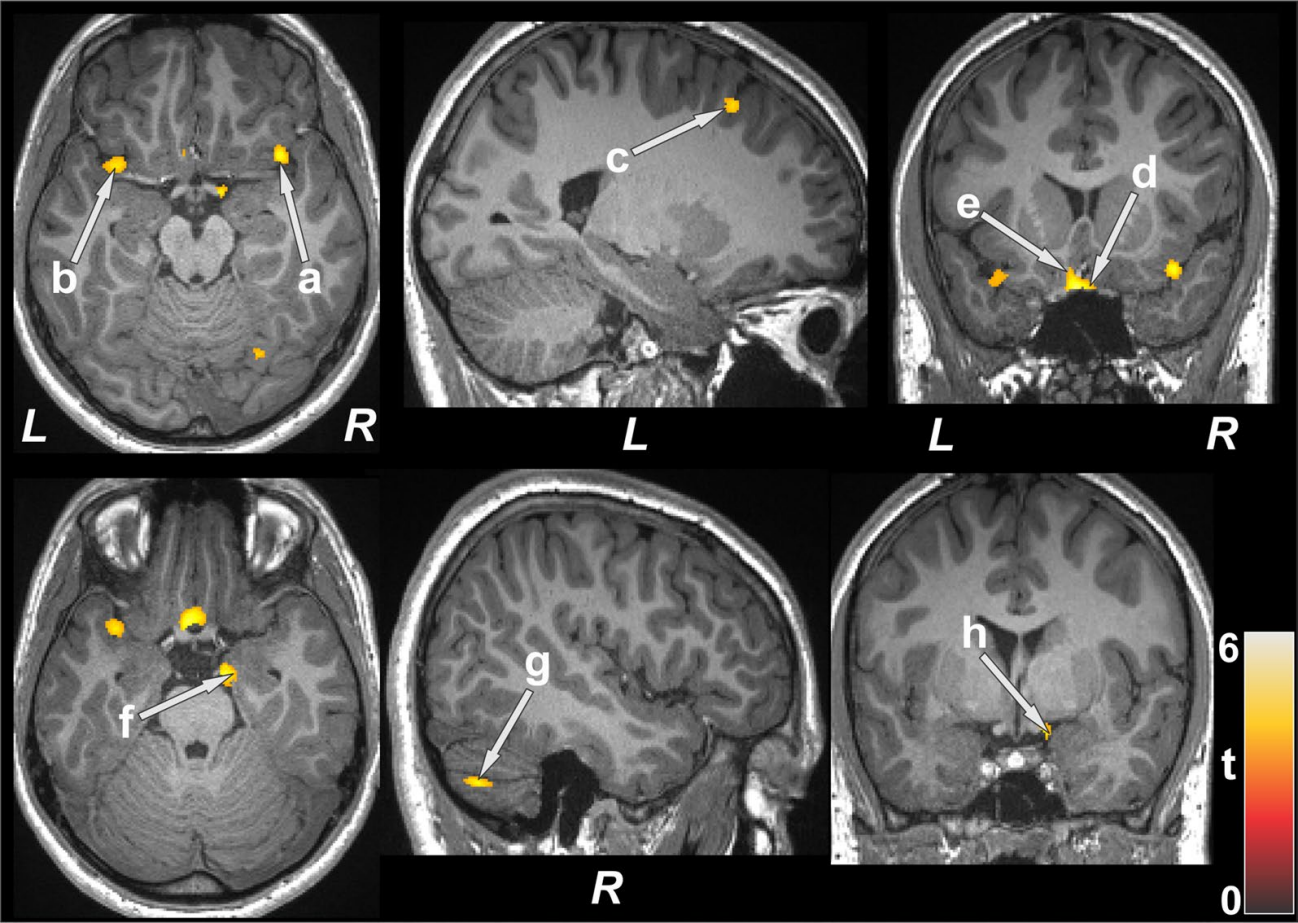
Brain regions with higher gray matter density in CF patients over control subjects. Sites with increased gray matter density included the bilateral insula (**a**, **b**), left frontal cortices (**c**), bilateral basal forebrain (**d**, **e**), right hippocampus (**f**), right cerebellum (**g**), and right amygdala (**h**). All images are in neurological convention (*L* left; *R* right; *M* middle). Color bar indicates t-statistic values

After controlling for age and sex, several brain areas showed increased GM density in CF subjects compared to healthy controls (Table 2). Very few sites emerged with significantly low GM density in CF compared to control subjects. Brain regions with increased GM density in CF subjects emerged in the right cerebellum, hippocampus, amygdala, parahippocampal gyrus, ventral medial prefrontal cortices, superior temporal cortices, bilateral basal forebrain, insula, parietal cortices, left mid and superior frontal, and prefrontal cortices, compared to controls (Fig. 1). Brain regions showing decreased GM density in CF patients emerged in the right inferior temporal cortices and bilateral occipital cortices.

**Table 2:** Regional brain gray matter density (mean ± SE, mm^3^/voxel) of CF patients and control corrected for age and sex

Brain areas	CF (n = 5)	Controls (n = 15)	p-values
	Mean ± SE (95% confidence interval)	Mean ± SE (95% confidence interval)	
L Prefr Cor	0.44 ± 0.013 (0.42–0.47)	0.38 ± 0.007 (0.37–0.40)	0.001
R Prefr Cor	0.47 ± 0.012 (0.44–0.49)	0.41 ± 0.007 (0.40–0.43)	0.001
R Vent Med Prefr Cor	0.57 ± 0.012 (0.54–0.59)	0.52 ± 0.006 (0.50–0.53)	0.002
L basal forebrain	0.49 ± 0.011 (0.47–0.52)	0.44 ± 0.006 (0.42–0.45)	< 0.001
R basal forebrain	0.45 ± 0.012 (0.43–0.48)	0.39 ± 0.007 (0.38–0.41)	0.001
R amygdala	0.38 ± 0.009 (0.36–0.40)	0.34 ± 0.005 (0.33–0.35)	0.001
R hippocampus	0.71 ± 0.006 (0.70–0.72)	0.68 ± 0.003 (0.68–0.69)	0.002
R parahippocampus	0.59 ± 0.007 (0.57–0.60)	0.55 ± 0.004 (0.54–0.56)	< 0.001
R cerebellar Cor	0.75 ± 0.010 (0.73–0.77)	0.70 ± 0.005 (0.69–0.71)	0.001
L insula	0.63 ± 0.015 (0.60–0.66)	0.56 ± 0.008 (0.55–0.58)	0.001
R insula	0.61 ± 0.013 (0.59–0.64)	0.55 ± 0.007 (0.54–0.57)	0.001
L mid frontal Cor	0.60 ± 0.007 (0.58–0.61)	0.55 ± 0.004 (0.55–0.56)	< 0.001
L sup frontal Cor	0.62 ± 0.011 (0.60–0.64)	0.57 ± 0.006 (0.55–0.58)	0.001
L sup parietal Cor	0.69 ± 0.019 (0.65–0.73)	0.61 ± 0.010 (0.58–0.63)	0.002
R sup parietal Cor	0.59 ± 0.016 (0.56–0.63)	0.51 ± 0.009 (0.49–0.53)	0.001
R sup temp Cor	0.67 ± 0.008 (0.65–0.69)	0.63 ± 0.005 (0.62–0.64)	0.001

*CF* cystic fibrosis, *SE* standard error, *L* left, *Prefr* prefrontal, *Cor* cortex, *R* right, *Vent* ventral, *Med* medial, *Mid* middle, *Sup* superior, *Temp* temporal

### Brain regions with T2-relaxation value differences

**Fig. 2:**
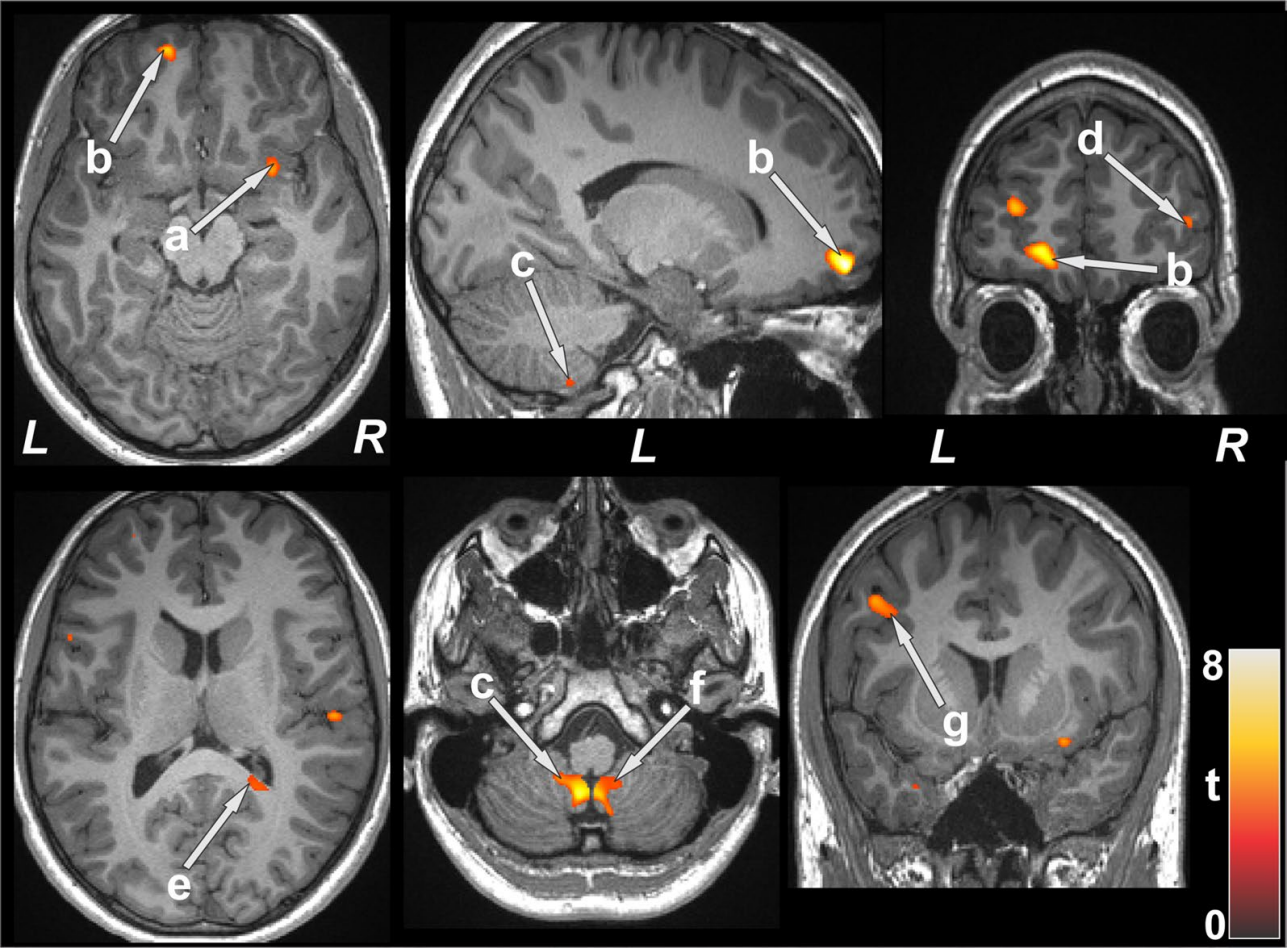
Brain sites with decreased T2 relaxation values in CF patients over control subjects. Reduced T2 relaxation values appeared in multiple regions and included in the right insula (**a**), bilateral prefrontal cortices (**b**, **d**), bilateral cerebellum (**c**, **f**), right corpus callosum (**e**), and left frontal cortices (**g**)

Several brain areas in CF participants showed significantly lower T2-relaxation values, indicating acute tissue injury, compared to control subjects (Table 3). Few brain sites showed significantly higher T2-relaxation values in CF compared to controls. Regions with significantly reduced T2-relaxation values in CF participants appeared in the bilateral cerebellum, cerebellar tonsil, prefrontal and superior temporal cortices, parietal cortices, left frontal cortices, and right insula (Fig. 2). Other sites, including white matter areas, were also detected with reduced T2-relaxation values in CF over controls in regions that link important gray matter regions associated with cognition, anxiety, and depression, including frontal white matter, corpus callosum, and medulla (Fig. 2). Brain regions showing prolonged T2-relaxation values in CF emerged in the bilateral hippocampus and left para-hippocampal gyrus.

**Table 3:** Regional brain T2-relaxation values (mean ± SE, ms) of CF patients and control corrected for age and sex

Brain areas	CF (n = 5)	Controls (n = 15)	p-values
	Mean ± SE (95% confidence interval)	Mean ± SE (95% confidence interval)	
L Prefr Cor	139.5 ± 3.3 (132.6–146.4)	162.5 ± 1.8 (158.7–166.3)	< 0.001
R Prefr Cor	162.6 ± 7.7 (146.2–179)	199.2 ± 4.3 (190.2–208.3)	0.001
L cerebellum	123.6 ± 4.3 (114.5–132.6)	149.3 ± 2.4 (144.3–154.3)	< 0.001
R cerebellum	123.3 ± 2.4 (118.1–128.4)	136.0 ± 1.3 (133.1–138.8)	< 0.001
L cerebellar tonsil	126.9 ± 4.8 (116.6–137.1)	160.8 ± 2.7 (155.2–166.5)	< 0.001
R cerebellar tonsil	127.5 ± 5.1 (116.7–138.3)	159.1 ± 2.8 (153.1–165)	< 0.001
R insula	157.6 ± 5.5 (146–169.1)	182.7 ± 3.0 (176.4–189.1)	0.001
Brainstem	138.1 ± 6.5 (124.4–151.8)	168.2 ± 3.6 (160.6–175.7)	0.001
L frontal Cor	133.8 ± 3.3 (126.9–140.8)	155.4 ± 1.8 (151.6–159.3)	< 0.001
L parietal Cor	152.4 ± 6.2 (139.2–165.6)	182.6 ± 3.4 (175.3–189.8)	0.001
R parietal Cor	128.0 ± 4.6 (118.4–137.7)	148.9 ± 2.5 (143.6–154.3)	0.001
L Sup Temp Cor	142.6 ± 4.8 (132.4–152.8)	166.0 ± 2.7 (160.4–171.6)	0.001
R Sup Temp Cor	132.1 ± 3.2 (125.3–138.9)	153.6 ± 1.8 (149.9–157.3)	< 0.001
Corpus callosum	121.3 ± 7.0 (106.4–136.2)	154.5 ± 3.9 (146.3–162.8)	0.001
L frontal WM	119.7 ± 2.4 (114.7–124.7)	134.2 ± 1.3 (131.5–137)	< 0.001

*CF* cystic fibrosis, *ms* millisecond, *SE* standard error, *L* left, *Prefr* prefrontal, *Cor* cortex, *R* right, *Sup* superior, *Temp* temporal, *WM* white matter

## Discussion

People with chronic diseases, such as CF, are at increased risk of depression and autonomic deficits. In addition, many aspects of the disease itself can lead to high levels of anxiety. We report significantly high scores of BAI and BDI-II in CF patients over healthy controls, consistent with previous studies [30, 31]. Also, we found that CF patients had a lower overall MoCA scores and this change was most significant in the visuospatial/executive sub domains. Several brain sites, including cerebellum, hippocampus, amygdala, insula, prefrontal, and temporal sites showed tissue changes based on GM density or T2-relaxometry procedures, areas that are involved in cognition, mood, and autonomic functions. Neuronal damage meditated through hypoxia and/or hypercapnia is considered to be one of the key mechanisms in pulmonary diseases [32, 33]. Both hypoxia and hypercapnia are often present in CF patients along with mutated CFTR gene and are potential underlying causes for the observed neural findings.

CF patients showed cognitive dysfunction, including the executive function, and mood deficits. Executive function is associated with skills requiring higher mental activities, such as setting goals, abstract logical thinking, planning, taking into account the long-term consequences, initiating intentional actions, creating different possible alternative reactions, or modifying own activity in response to changing conditions. Abnormal executive function has been found in other diseases with respiratory compromise, such as chronic obstructive pulmonary disease, asthma, obstructive sleep apnea [34–36], and abnormal function in CF patients may contribute to such diminished actions. Depression and anxiety, as observed in our study, affects disease management, including clinic attendance and adherence to prescribed treatments, leading to increased hospitalization and healthcare costs, worse pulmonary function, and decreased health-related quality of life [31, 37–39]. These findings reiterate the need for annual screening for depression and anxiety in patient with CF.

Although cognitive and mood symptoms are considered to be due to aspects surrounding the diagnosis of the disease, our findings show that CF patients have a brain structural basis for these symptoms. CF patients showed increased GM density and reduced T2-relaxation values in several brain areas, though GM density measures indicated more changes over T2-relaxometry. Such particular brain tissue changes were evident in the cerebellum, hippocampus, amygdala, superior temporal cortices, basal forebrain, insula, parietal cortices, and frontal and prefrontal cortices. The increased GM density or reduced T2-relaxation values in our patient population might be due to increased neuronal and axonal swelling (although the disease is chronic, the condition is associated with ongoing hypoxia), increased size neurons, increased glial cell size or number, higher vascular density to support sustained increased metabolic demand, more connective tissue, dendritic outgrowth, or synaptogenesis [40, 41]. Higher neuronal numbers may result from an abnormal developmental process, including accentuated neuronal birth rate or the survival of excess neurons [40]. In addition, the elevated GM density may be related to pre-apoptotic osmotic changes or hypertrophy, marking areas of early neuronal deficits. Previous depression studies indicated increased glucose metabolism [42, 43] resulting from the inhibition of reciprocal connections between the prefrontal cortex and the amygdala in limbic-thalamic-cortical circuit or limbic-cortical-striatal-pallidal-thalamic circuit enlarging amygdala [41, 44], and such processing may be operating in other structures as observed here.

The hippocampus, prefrontal cortices, and amygdala regions are highly interconnected and constitute the neuroanatomical network for mood regulation [44, 45], and these areas showed increased GM density or altered T2-relaxation values in our study. Activation of the amygdala has been demonstrated to increase dopamine in the nucleus accumbens and other motor control centers, resulting in increased fear behaviors and anxiety and might be plausible explanation for higher anxiety in CF patients. The amygdala and hippocampus have projections from the prefrontal cortices and other limbic-related forebrain structures that are involved in several cognitive domains, and increased GM volume or altered T2-relaxation values in these sites, as found in our study, may suggest abnormal cognition. The superior temporal gyrus has connections to limbic and prefrontal regions [46], and right superior temporal structures in particular have been associated with responses to emotional prosody [47]. The superior temporal lobe along with insula and cingulate regions form a part of the salience network that is involved in the coordination of the behavioral responses. The anterior insula displays altered functional connectivity within the salience network and with other brain network in depression condition. Another brain region that showed increased GM density and tissue changes was cerebellum, where climbing fiber codes error signal reflecting the motor performance failure and works to depress the synaptic transmission between parallel fibers and Purkinje cell that can lead to depression and autonomic deficits [48, 49]. Furthermore, the cerebellum contributes to cognitive processing in several cognitive domains, including executive and visuospatial functioning and extensively interconnected with the cerebral hemisphere, both in feed-forward and feed-backward directions, and provides a structural basis for cognitive deficits in CF patients.

Patients with CF experience a wide spectrum of chronic pain, including headache, chest pain, back pain, abdominal pain, and limb pain [50]. Brain regions that showed increased GM density or altered T2-relaxation values, including the insula and hippocampus, are subjected to pain modulation and stress-induced changes [51, 52]. Stress can lead to microglial proliferation in areas around the third ventricle, including hippocampus, and activate microglia that can cause neuronal damage with the release of proinflammatory and cytotoxic factors and plausibly increase GM density or alter T2-relaxation values as observed in our study. Several brain areas showed reduced T2-relaxation values in CF patients which could result from increased astrocyte and microglial activation due to chronic pain. Earlier human postmortem studies reported reduced T2-relaxation values due to pronounced reactive microgliosis and astrogliosis [13], and showed the association between chronic pain and prolonged astrocyte activation at the level of the primary afferent synapse [53, 54].

Multiple diseases have demonstrated altered GM and white matter volume and tissue integrity [14–16, 55, 56]. However, this is the first study that shows significant brain structural (GM density and brain tissue integrity) changes in CF patients, which could account for the symptomatology reported in the condition. Several basic and clinical studies, particularly those using neuroimaging techniques, report that specific brain regions play essential roles in cognitive, autonomic, depression, and anxiety regulation [55, 56]. The altered brain regions we encountered in CF patients have a considerably important role in their cognitive and mood wellbeing. With a high incidence of psychological symptoms in adult CF patients, this study highlights the importance for improved early identification and management strategies for adult CF patients.

One of the limitations of this study is the small sample size that may affect the statistical analyses with findings not corrected for multiple comparisons, and may limit the magnitude of the significant alterations, as well as with type 1 error that we observed in various brain regions of CF patients. Therefore, further studies are needed to validate these findings. T2-relaxometry procedures had poor resolution in slice thickness direction, resulting to less sites with damage over GM density measures. Thus, procedures with higher resolution would be required with bigger sample size to examine the extent of tissue damage. We used MoCA, BDI-II, and BAI screening instruments to identify cognitive impairment and symptoms of depression and anxiety, combined with comprehensive clinical tests should be used for future studies.

## Conclusions

Patient with CF showed significant brain structural changes, as evidenced by altered GM density or T2 relaxation values, indicative of tissue injury, in brain regions that control cognitive, autonomic, and mood functions. These sites included the cerebellum, hippocampus, amygdala, superior temporal cortices, basal forebrain, insula, parietal cortices, frontal and prefrontal cortices, and corpus callosum. In addition, CF patients exhibited significant anxiety and depression symptoms and impaired cognitive abilities, and brain regions regulating such functions showed altered brain structural integrity. Integration of mental health screening and early identification and targeted treatment of CF patients can improve the mortality and morbidity seen in the condition.

## Data Availability

The datasets used and/or analysed during the current study are available from the corresponding author on reasonable request.

## Abbreviations

ANCOVA: Analysis of covariance
BAI: Beck anxiety inventory
BDI-II: Beck depression inventory-II
CF: Cystic fibrosis
CFTR: Cystic fibrosis transmembrane conductance regulator
FA: Flip angle
FOV: Field of view
GM: Gray matter
MNI: Montreal neurological institute
MoCA: Montreal cognitive assessment
MRI: Magnetic resonance imaging
PD: Proton density
SI: Signal intensity
SPM12: Statistical parametric mapping 12
TE: Echo-time
TR: Repetition time
VBM: Voxel-based morphometry
